# Mining breast cancer genes with a network based noise-tolerant approach

**DOI:** 10.1186/1752-0509-7-49

**Published:** 2013-06-25

**Authors:** Yaling Nie, Jingkai Yu

**Affiliations:** 1 National Key Laboratory of Biochemical Engineering, Institute of Process Engineering, Chinese Academy of Sciences, Beijing 100190, China

**Keywords:** Network, Breast cancer, Data noise, Noise tolerance

## Abstract

**Background:**

Mining novel breast cancer genes is an important task in breast cancer research. Many approaches prioritize candidate genes based on their similarity to known cancer genes, usually by integrating multiple data sources. However, different types of data often contain varying degrees of noise. For effective data integration, it’s important to design methods that work robustly with respect to noise.

**Results:**

Gene Ontology (GO) annotations were often utilized in cancer gene mining works. However, the vast majority of GO annotations were computationally derived, thus not completely accurate. A set of genes annotated with breast cancer enriched GO terms was adopted here as a set of source data with realistic noise. A novel noise tolerant approach was proposed to rank candidate breast cancer genes using noisy source data within the framework of a comprehensive human Protein-Protein Interaction (PPI) network. Performance of the proposed method was quantitatively evaluated by comparing it with the more established random walk approach. Results showed that the proposed method exhibited better performance in ranking known breast cancer genes and higher robustness against data noise than the random walk approach. When noise started to increase, the proposed method was able to maintained relatively stable performance, while the random walk approach showed drastic performance decline; when noise increased to a large extent, the proposed method was still able to achieve better performance than random walk did.

**Conclusions:**

A novel noise tolerant method was proposed to mine breast cancer genes. Compared to the well established random walk approach, it showed better performance in correctly ranking cancer genes and worked robustly with respect to noise within source data. To the best of our knowledge, it’s the first such effort to quantitatively analyze noise tolerance between different breast cancer gene mining methods. The sorted gene list can be valuable for breast cancer research. The proposed quantitative noise analysis method may also prove useful for other data integration efforts. It is hoped that the current work can lead to more discussions about influence of data noise on different computational methods for mining disease genes.

## Background

Novel disease genes remain difficult to identify in most genetic diseases, and in particular, in highly polygenic disorders. Currently, not all genes have yet been detected even for those diseases whose molecular mechanisms are partially known [1], for instance, breast cancer [2]. Breast cancer is a common cancer and a major cause of cancer death among females around the world, which makes up 23% of total cancer cases and 14% of cancer deaths [3]. Mining breast cancer genes is conducive to understand its pathogenic mechanism and search for effective treatments. With rapid growth of disease-related genomic and functional data, computational approaches can be utilized to mine for new cancer genes [4].

In the past two decades, a number of computational methods had been developed to mine potential disease related genes. Most of those methods rank candidate genes based on the idea that proteins similar to each other tend to cause similar or same diseases [5]. They involve setting up a candidate gene set to be compared with a known disease gene set on their physical or functional attributes [6]. On one hand, physical attribute-based methods include screening direct neighbors of known disease genes in the PPI network [7,8], comparing shortest path length [9] between candidate genes and known disease genes, clustering or graph partitioning to uncover disease modules in the interaction network [10-12]. Some approaches also used global network features to find genes similar with known disease genes [13,14]. On the other hand, several methods rely on functional similarities between candidate and disease genes [15], for example, some methods measured similarity between genes by their functional annotations [16] (e.g., Gene Ontology (GO) [17]). Methods using other data sources had also been developed, such as gene expression [18,19], biological pathways and sequence features [20].

Cancers such as breast cancer are complex and heterogeneous in nature, cancer-related genes often do not function in isolation but interact with one another [5]. Integrating multiple data types was found to be effective for gene mining in alleviating problems caused by incomplete information [21-23]. For instance, ENDEAVOUR [24] is an online tool based on using multiple data sources. It integrated candidate gene rankings from different data sources into a final ranking with the order statistic algorithm. However, different data categories usually contain inherent noise or systematic errors [25]. For instance, data from computational predictions will no doubt contain some amount of uncertainty. Experimental data obtained from different labs or experimental platforms can contain appreciable amount of noise. Noise in source data can push computed results away from their true values, lead to erroneous reporting.

A better method must be able to tolerate certain amount of noise, which makes the integration of different data sources more applicable to real-life scenarios. Despite the fact that some approaches can work with precision when presented with highly accurate data, few studies have shown that those methods worked robustly when faced with increasingly noisy data. A number of papers had discussed the task of balancing noise and precision when using multiple data sources for cancer gene mining, however, hardly anyone had analyzed the noise problem quantitatively [26-29]. It is important to calibrate how robust a method works with respect to noise, namely, how fast a method deteriorates when percentage of noise in source data goes up. With that knowledge, users can then be confident about the method’s effectiveness when it is applied to real life data sets.

**Figure 1:**
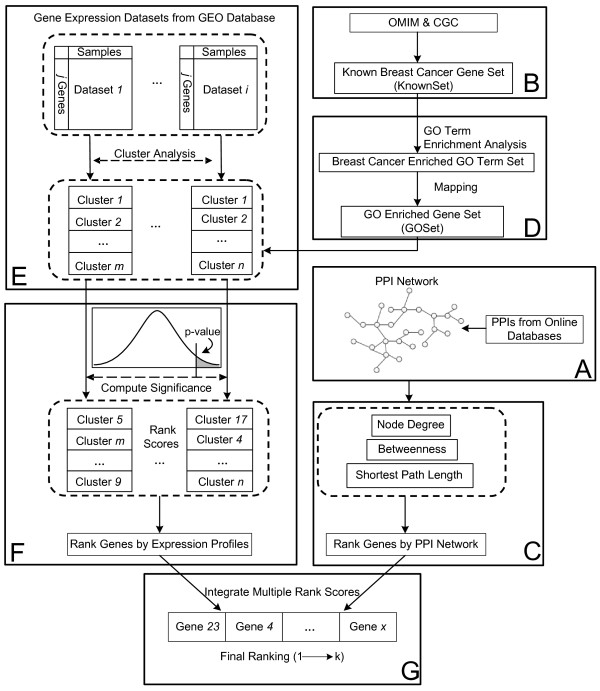
**Schematic chart for mining breast cancer genes.** Four different types of data were used as input: PPI data, human gene expression data, known breast cancer genes and GO annotations. Gene expression data (GDSes) from the GEO database were clustered. Known breast cancer genes and their enriched GO annotations were used to rank genes in those clusters. From the PPI network, three network topological attributes were computed to rank genes in the network. Finally, all individual rankings were combined into a final rank, which represents a gene’s overall probability of being involved in breast cancer.

To tackle the data noise problem, a novel noise tolerant data fusion approach was proposed here for breast cancer gene mining (Figure 1), which integrated information from PPI network with gene expression data to rank genes based on their probabilities of being breast cancer related. Satisfactory results were obtained even when noise level was high. To demonstrate advantages of the proposed method, its performance was compared with that of the random walk method [13], which utilized a node’s global neighborhood in a network to rank genes. Random walk based methods had been shown to produce good performance [18,23,29-31] in gene ranking. Results showed the proposed method exhibited better robustness when faced with increasingly noisy data, as compared to the random walk approach.

## Results and discussion

An effective data integration method was developed to mine breast cancer genes from four major data sources: Protein-Protein interactions, gene expression data, GO annotations, and known breast cancer genes (Table 1).

**Table 1:** Data source

**Data categories**	**Volume of input data**	**Original sources/tools**	**Volume of original data**	**Download date**
**PPI network**	156,459 PPIs	HPRD	39,240 PPIs	Mar. 3, 2013
		BioGRID	129,180 PPIs	Mar. 3, 2013
		homoMINT	33,502 PPIs	Mar. 3, 2013
		IntAct	95,746 PPIs	Mar. 3, 2013
		Human Signalling Network	59,111 PPIs	Mar. 3, 2013
**Gene expression data**	53 GDSes	GEO	57 GDSes	Apr. 7, 2011
**Known cancer genes**	37 genes	OMIM	30 genes	Mar. 3, 2013
		CGC	19 genes	Mar. 3, 2013
**GO term (BP)**	80 terms	DAVID*	50 terms	Mar. 3, 2013
		GOEAST*	50 terms	Mar. 3, 2013
		GOstats*	50 terms	Mar. 3, 2013
		Cancer-hallmark GO terms	9 terms	Mar. 3, 2013

* For the known breast cancer gene set, three tools were used to perform the enrichment analyses of GO terms in the BP sub-ontology: DAVID (http://david.abcc.ncifcrf.gov/home.jsp), GOEAST (http://omicslab.genetics.ac.cn/GOEAST/), and GOstats (R package in Bioconductor). DAVID and GOEAST are web tools; GOstats is an R package from Bioconductor. Cancer-hallmark GO terms were extracted from Table 1 of [37].

After removing redundancy, a comprehensive human PPI network was constructed with data obtained from multiple interactions databases. The resultant network contained a total of 156,459 PPIs with 15,494 genes. A noise tolerant method was designed to rank potential breast cancer genes.

### Rationale for data integration

**Figure 2:**
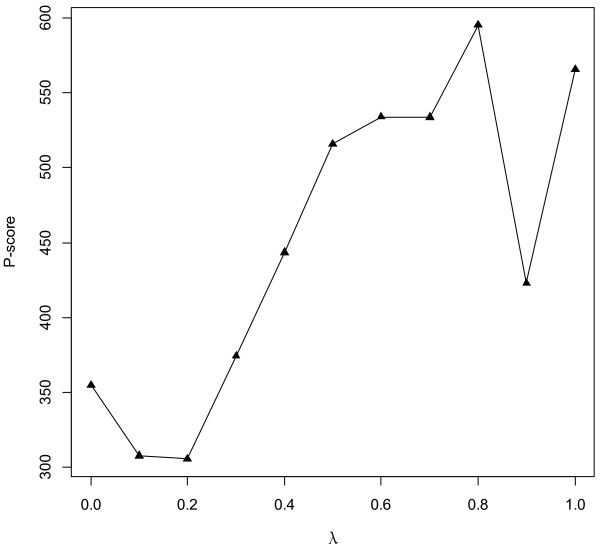
**Performance of our method for different *λ *values.** P-score was the average ranking of KnownSet in top 10% of the final sorted list. A smaller P-score (ranked higher) meant better capability to correctly rank known breast cancer genes.

A network-based score (*S*_*N*_) and an expression-based score *(S*_*E*_) were respectively derived for each gene, which were then integrated into a final score (*S*) by weighting them with a coefficient *λ*. A P-score was computed to represent performance of the proposed method when *λ* was changed from 0 to 1. P-score was the average ranking of known breast cancer genes in top 10% of the final gene ranking list (see Methods). Smaller P-score (ranked higher) meant better performance (Figure 2). As shown in Figure 2, better performance was achieved when *λ*=0 (i.e., using only network-based ranking) compared with *λ*=1 (i.e., using only expression-based ranking); the best performance was obtained when *λ*=0.2, which suggested that utilizing genes’ complex relations in the PPI network can help cancer gene mining tasks. It also confirmed that multiple data sources can complement each other in ranking cancer genes. Final ranking results were listed in Additional file 1: Table S1.

### Evaluation of performance in ranking known cancer genes

Known cancer genes were derived from the OMIM (Online Mendelian Inheritance in Man) and CGC (Cancer Gene Census) databases (see Methods). Known breast cancer genes derived from OMIM was called OMIMSet, those from CGC called CGCSet. OMIMSet was used to train the proposed method, and CGCSet was used for evaluation. Table 2 showed that the proposed method achieved better performance in ranking known breast cancer genes. Counting only genes ranked in top 10%, the proposed method achieved an average ranking of 279, compared with 545 by random walk. Counting all 11 genes in CGCSet, the proposed method achieved an average ranking of 1801, compared with 2207 by random walk.

**Table 2:** Ranking performance comparison

** The proposed method**		** Random walk approach**	
**Gene**	**Ranking**	**Gene**	**Ranking**
RB1	*89*	RB1	*60*
CCND1	*105*	EP300	*119*
EP300	*142*	CCND1	*227*
ERBB2	*171*	ERBB2	*326*
MAP2K4	*450*	NTRK3	*685*
GATA3	*463*	MAP2K4	*1167*
BAP1	*530*	BAP1	*1231*
PBRM1	1626	GATA3	3042
ETV6	2952	PBRM1	3102
NTRK3	4030	ETV6	3355
SLC22A18	9254	SLC22A18	10971
***top 10% average***	*279*		*545*
**all 11 test genes average**	1801		2207

### Robustness with respect to realistic data noise

**Figure 3:**
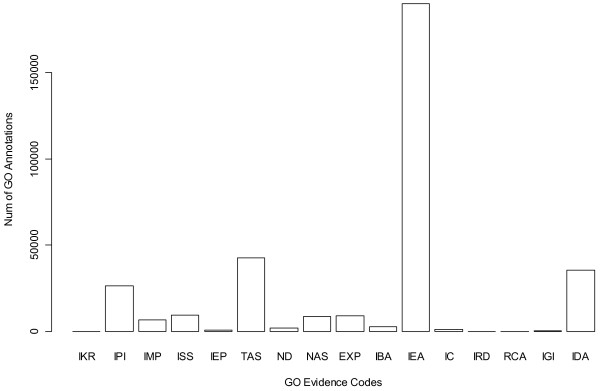
**Evidence code distribution of GO annotation (for *Homo sapiens*).** IEA means that the denoted GO annotations were inferred from electronic annotation and not curated manually. IEA counted for the majority of GO annotations.

In general, a method based on multiple types of knowledge is more objective than those utilizing singular information; combining independent data sources can alleviate effects of biases inherent in single data types [32]. Most data sets, especially genome wide data, tend to contain appreciable amount of noise. For instance, GO is a powerful tool which provides a controlled vocabulary to describe biological functions on multiple levels [33]. It was also widely used on cancer research (there are about 39,000 citations for GO in Google Scholar by Oct. 16, 2012). However, not all GO annotations are equally credible [34]. As of October 2012, there were over 3 million GO annotations for *Homo sapiens* genes. Each GO annotation includes an evidence code to indicate how the annotation was inferred. All evidence codes (except IEA, which is automatically derived) are manually assigned. They can be divided into four categories: experimental (EXP, IDA, IPI, IMP, IGI, and IEP), computational analysis (ISS, ISO, ISA, ISM, IGC, IBA, IBD, IKR, IRD, and RCA), author statements (TAS and NAS), and curatorial statements (IC and ND). For *Homo sapiens*, 16 evidence codes were used to describe GO annotations [35]. The vast majority of GO annotations were found to be computationally derived and not manually curated (IEA evidence code) (Figure 3). GO annotations therefore contain appreciable amount of noise within themselves.

Approaches that exhibit robust performance with regard to noise are needed if they are to prove useful in cancer gene hunting endeavours. Nevertheless, as mentioned before, few projects had specifically analysed data noise effects quantitatively. A network based noise tolerant method was proposed here to mine breast cancer genes. Its performance was compared with that of the well performing random walk approach by five-fold cross-validation. The results confirmed the proposed method’s robust performance with respect to data noise.

The set of known breast cancer genes (KnownSet, see Methods) was enlarged by including genes sharing GO annotations with those in the KnownSet. The enlarged set was called the GOSet (GO enriched gene set, see Methods), which was adopted as a noisy set of likely breast cancer genes. The GOSet was utilized to check an algorithm’s robustness with respect to data noise. Data were sampled from the GOSet, and combined with the KnowSet to generate a noisy set of training data. This way of synthesizing noisy data set is unique in that it doesn’t simply using random data as noise, which is too artificial. The GOSet contains enriched but still imperfect data, which can better mimic data noise in real life scenarios. An algorithm’s ability to retain its performance was checked when fraction of noisy data in the training set went up.

**Figure 4:**
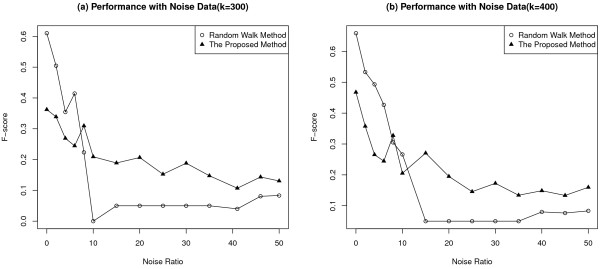
**Five-fold cross-validation performance evaluation.** In cross validation, different ratios of noise data were added to input data, with the ratio changing from 0 to 50, where 0 meant only known breast cancer genes were used as input data. Performance was evaluated in terms of the F-score. *k* was a ranking threshold to judge a ranked gene as a true breast cancer gene by the proposed method. **(a)***k*=300; **(b)***k*=400.

The proposed method did not work quite as well as random walk approach when input data was 100% accurate, however, when noise level in input data increased, we observed the following phenomena (Figure 4).

1. Random walk approach exhibited a sharp decrease in its performance, while our method was able to maintain a relatively stable performance.

2. When noise increased to a large extent, the proposed method was able to perform about twice as well as random walk approach did.

It can thus be stated that the proposed method was more robust with respect to noise in input data, compared to the state-of-art random walk based approach. The results also confirmed the power of data integration, which was able to let different data sets complementing each other [22,23].

### Robustness with respect to completely random noise

**Figure 5:**
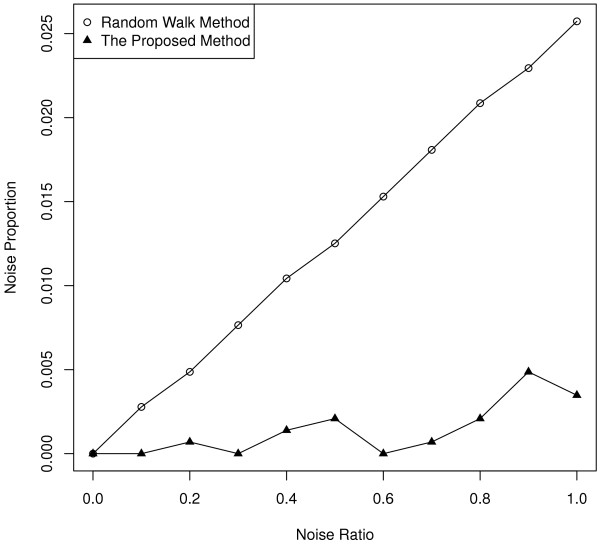
**Ranking comparison performance.** Randomly picked genes were added to the KnownSet, and performances of the proposed method and random walk approach were then compared.

GOSet tried to simulate realistic data noise. However, it might be suspected that GOSet was biased toward the proposed method in one way or another. To make sure the comparison between the proposed method and the random walk approach was not done unfairly, randomly picked genes were added to the KnownSet (Figure 1, Box B, see Methods), and performances of the proposed method and random walk approach were then compared. Figure 5 again showed that random walk approach showed linear performance decline when noise increased in the source data, while the proposed method was able to maintain stable performance.

## Conclusions

Cancers are highly complex processes, the majority of cancer genes are yet to be mapped. Currently available data (known breast cancer genes) are too limited to be really effective for cancer gene searching purpose. Broadening the scope of input data (both volume and type) should enable better use of available data to mine for new cancer genes. Approaches that work robustly against data noise are needed.

A novel noise tolerant breast cancer gene mining method was presented here, which integrated a comprehensive PPI network, gene expression data, prior knowledge of breast cancer and GO annotations to rank potential breast cancer genes. From each data source, a ranked list for each candidate gene was computed, and they were then combined into a final ranking order. Influence of data noise was quantitatively evaluated. Random walk approach performed better than the proposed method using 100% accurate input data (known breast cancer genes). However, the proposed method showed much greater noise tolerance. To our best knowledge, this is the first effort to quantitatively analyse noise tolerance between different cancer gene mining methods. The framework of the proposed mining method and the quantitative way of appraising noise effects are flexible enough to be useful for other data sources, and hopefully, lead to more discussions on data noise issue for different computational methods in cancer gene mining field.

## Methods

Figure 1 presented a schematic view of our approach. A comprehensive PPI network was obtained by integrating data from different interactions databases [36] (Box A). A set of known breast cancer genes (KnownSet) was extracted from the OMIM and CGC databases (Box B). Candidate genes were first ranked by three network topological attributes: node degree, node betweenness and by their closeness to known cancer genes in the network (Box C). GO term enrichment analyses were performed for KnownSet, producing a GO term set enriched with breast cancer related terms, into which a group of cancer-hallmark GO terms were also added [37]. A set of genes which were annotated with terms in the obtained GO set were generated, which was called the GO enriched gene set (GOSet) (Box D). A batch of breast cancer-related expression data was extracted from the GEO database [38] on April 7, 2011 and expression profiles in those data files were clustered based on their similarity with each other (Box E). Expression clusters were intersected with GOSet. Overlap significance was represented by a p-value computed with the normal distribution. The p-value was utilized to rank genes in expression clusters (Box F). All individual rankings from different data sources were finally combined into a final ranking, which represented a gene’s overall probability of being involved in breast cancer (Box G).

### Deriving known breast cancer gene set

Thirty known breast cancer genes were extracted from the OMIM database [39] and 19 from the CGC database [40] (Table 3). For a gene to be usable, it was required to be covered by both the PPI network and expression data sets. With that requirement, 26 genes derived from OMIM (OMIMSet) and 11 additional genes from CGC (CGCSet) were obtained, this set of 37 genes was called the KnownSet.

**Table 3:** The known breast cancer genes

**Symbol**	**NCBI_ID**	**Symbol**	**NCBI_ID**
AKT1^1,2^	207	RAD51^1^	5888
AR^1^	367	RAD51C^1^	5889
ATM^1^	472	TP53^1,2^	7157
BARD^1^1	580	TSG101^1^	7251
BRCA1^1,2^	672	XRCC3^1^	7517
BRCA2^1,2^	675	RAD54L^1^	8438
RAD51D^1,3^	5892	PPM1D^1^	8493
CASP8^1^	841	RB1CC1^1^	9821
CDH1^1,2^	999	CHEK2^1,2^	11200
NQO1^1^	1728	PALB2^1,2^	79728
ESR1^1^	2099	BRIP1^1,2^	83990
HMMR^1^	3161	BCPR^1,3^	8142
KRAS^1^	3845	BRCATA^1,3^	8068
NQO2^1^	4835	SLC22A18^1^	5002
PHB^1^	5245	BRCA3^1,3^	60500
PIK3CA^1,2^	5290	ERBB2^2^	2064
BAP1^2^	8314	ETV6^2^	2120
CCND1^2^	595	GATA3^2^	2625
EP300^2^	2033	MAP2K4^2^	6416
PBRM1^2^	55193	NTRK3^2^	4916
RB1^2^	5925		

^1^ known breast cancer genes from OMIM database.

^2^ known breast cancer genes from CGC database.

^3^ known breast cancer genes that could not be mapped to the PPI network or the GDSes.

### Ranking by PPI network

The human PPI data were derived from five sources: HPRD [41], BioGRID [42], homoMINT [43], IntAct [44] and a manually curated human signalling network [45]. Protein identifiers were mapped to uniform coding gene identifiers. Official gene symbols were used as identifier. Redundant interactions were removed, along with interactions with identifiers that could not be mapped to gene symbols (Table 1). The final PPI network was represented by an undirected graph where nodes representing genes and edges representing interactions. The graph contained 156,459 interactions connecting 15,494 genes.

Similarities between proteins were found to be correlated with their proximity in the PPI network [46]. It was assumed that when a gene in the PPI network exhibited topological features similar to known breast cancer genes, it’s more likely to be involved in breast cancer processes. Several papers had shown that cancer genes could be effectively distinguished from others by their topological attributes in the PPI network, such as node degree [47], betweenness centrality [48] and shortest path length [10]. The above three network topological indices were computed and used to assess gene similarity in the PPI network. Genes were then sorted according to values of the topological indices.

Let **G**(**V**,**E**) be the PPI network, where **V** is the set of genes, and **E** the set of interactions in the network.

For a node *v*∈**V**, **degree***c*_*d*_(*v*) is the number of direct neighbours of *v* in the network. **Betweenness centrality** is the sum of the fraction of all-pair shortest paths that pass through *v*[49]. It can be expressed as following,

(1) $$c_{b}\left(v\right)=\sum_{j,k\in V}\frac{\sigma_{\mathrm{jk}}\left(v\right)}{\sigma_{\mathrm{jk}}}$$

where σ_*jk*_ is the number of shortest paths from a source *j*∈**V** to a target *k*∈**V**, and σ_*jk*_(*v*) is the number of those paths passing through some node *v* other than *j*,*k*. If *j*=*k*, σ_jk_ =1, and if *v*∈*j*,*k*, σ_*jk*_(*v*)=0.

The **shortest path length** was defined as the average shortest path distance from all known breast cancer genes to node *v*, it can be denoted as following,

(2) $$\frac{c_{\mathrm{spl}}\left(v\right)=\sum_{t\in G}d\left(v,t\right)}{n}$$

where **G** is the KnownSet, d(*v*,*t*) is the shortest path length between node *v* and *t. n* is the number of known breast cancer genes which can be reached by *v*.

**Figure 6:**
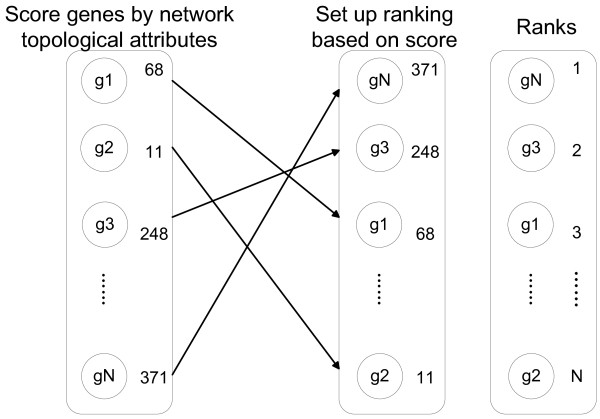
**Ranking genes by network attributes.** Genes of the PPI network were sorted according to network attributes (node degree was used as an example here). The scores were then converted into ranking values.

The above topological attributes were computed with the Python package networkx [50]. To facilitate later integration step, they were transformed into rankings; that is, each list was sorted, and a gene was assigned a positive integer number for a specific attribute according to that attribute’s value in the sorted list (Figure 6). In the end, three rankings for each gene were obtained based on its topological attributes in the network. *S*_*N*_(*v*) was the topology-based ranking score of a gene *v*, which was calculated from the three network topological attributes: **node degree**, **shortest path length** and **node betweenness**, *S*_*N*_(*v*) = (*c*_*d*_(*v*) + *c*_*spl*_(*v*) + *c*_*b*_(*v*))/3.

### Deriving GO enriched gene set

**Figure 7:**
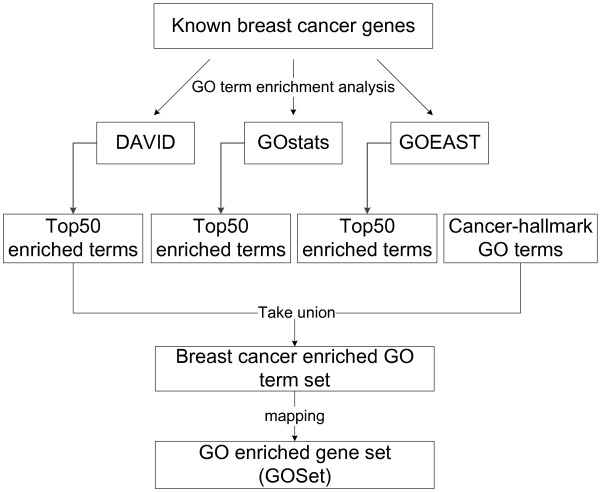
**Getting GO enriched gene set by GO enrichment analysis.** Three different tools were used to perform GO term enrichment analyse for known breast cancer genes. Top 50 enriched GO terms were picked from results obtained by each tool, and their union were generated. Nine cancer-hallmark GO terms from [37] were added into the enriched GO term set. The enriched GO term set were re-mapped back to a set of human genes based on Homo sapiens GO annotations, called the GO enriched gene set (GOSet).

GO provides a controlled vocabulary of terms for describing genes and gene products [17]. GO enrichment analysis assesses whether certain GO annotations are significantly over represented among a set of genes [51]. The level of enrichment can be represented by a p-value based on specific probability distribution. The p-value is calculated by randomly picking sets of genes from the genome and computing the probability of obtaining more genes with GO terms annotated to those in the study set [52]. The smaller the p-value, the more significant the GO term is enriched in the gene set. For the KnownSet, we used three tools (DAVID [53], GOstats [54], GOEAST [55]) to perform enrichment analyses in the BP (Biological Process) sub-ontology. DAVID and GOEAST are online tools and GOstats is an R package from Bioconductor [56]. The top 50 enriched terms were picked from results obtained by each of those tools. The three enriched GO term sets from the three tools were combined into one GO term set by taking their union. In addition, cancer-hallmark related GO terms are those characteristically related to cancers, they should also be included. The set of cancer-hallmark GO terms listed in Table 1 of [37] were added to the above obtained GO term set, which was then remapped to a set of corresponding genes based on human GO annotations. Genes not covered by our PPI network were removed. The obtained set of genes was enriched with BP terms annotated to known breast cancer genes; they thus were more likely to be involved in breast cancer than randomly selected genes. This set of genes was called the GO enriched gene set (GOSet) (Figure 7).

### Ranking by gene expression and GO

All breast cancer-related gene expression datasets (keywords: *Homo sapiens* & breast cancer) were download from the GEO database [38]. Data sets with fewer than five samples or conditions were deleted. Data sets of normal versus cancer samples were used so those containing recurred versus non-recurred samples were deleted. 53 GDSes (GEO data sets) were thus obtained.

For each GDS, records with “*null*” information and genes which didn’t exist in the PPI network were removed, and if a gene had more than one expression profiles, its expression was defined as the profile which had the largest mean value [57], defined as:

(3) $$E\left(i\right)=\max_{k\in n}\left(\frac{\sum_{j=1}^{m}e_{k}\left(j\right)}{m}\right)$$

where *n* is the set of expression profiles for gene *i* in a GDS, *m* is the number of samples/conditions in one of those profiles, and *e*_*k*_(*j*) is the corresponding expression value of sample *j*.

After the above mentioned preprocessing steps, genes in each GDS were clustered by the APCluster algorithm according to their expression profiles. APCluster is an algorithm based on affinity propagation which works by considering all data points as potential cluster centers at the same time and setting up messages of similarity between any two data points, messages are exchanged among data points until all clusters are determined. APCluster had been shown to perform well compared to other clustering approaches [58,59]. Pearson correlation coefficient between gene expression profiles was used as the similarity metric for APCluster. It was assumed that genes within a cluster would have higher probability of being involved in certain biological processes than those across clusters.

**Figure 8:**
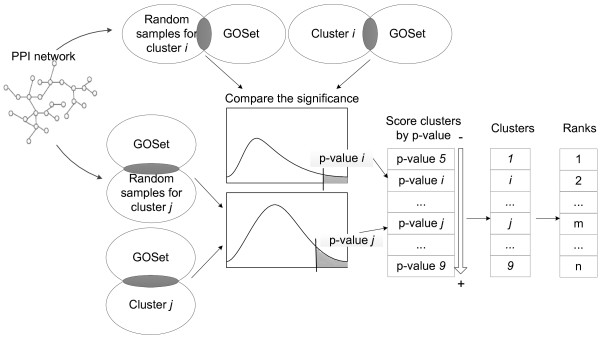
**Ranking gene clusters from GEO expression profiles.** For a cluster *i*, random samples of same size were drawn from the same GDS and their overlaps with GOSet were computed. P-value was used to represent significance of a cluster’s overlap with GOSet. The smaller the p-value, the higher the ranking.

Overlaps of expression clusters with the GOSet were next computed. To evaluate significance of the overlaps, the same number of genes was randomly sampled as those in the cluster from GDS, and its overlap with the GOSet was computed; the procedure was repeated 1000 times for each cluster. A p-value was then computed for the clusters (Figure 8). Rankings were assigned to clusters according to their p-values; that is, the lower the p-value, the higher the position in the ranking list. Genes in the same clusters were assigned the same ranking. A ranking score *S*_*E*_(*i*) was thus obtained for genes in each GDS, where *i* represented a specific GDS. A score S_*E*_ was assigned for each gene by computing the average of S_*E*_(*i*) from all relevant GDSes.

### Ranking integration

For a gene *v*, the ranking scores based on network topology and expression clustering were combined into an overall ranking as following,

(4) $$S\left(v\right)=\left(1-\lambda \right)S_{N}\left(v\right)+\lambda S_{E}\left(v\right)$$

*S*_*E*_(*v*) was the expression-based ranking score of gene *v*, which was computed from breast cancer-related gene expression data and GO annotations. *λ* (0≤*λ*≤1) is a coefficient to weigh the contribution of topological attributes and expression information in ranking breast cancer genes. The average ranking of genes in the KnownSet that sorted into top 10% was computed as the P-score. A smaller P-score meant better performance, that is, it was more likely to find true breast cancer genes from the top of the sorted list. *S*(*v*) is the final ranking for a gene *v,* which reflected the belief that a specific gene was a potential breast cancer gene. The higher a gene was ranked, the more likely it was involved in breast cancer related processes.

### Random walk approach

The steps in [13] were followed to perform random walk. It iteratively transitions a walker from its current node to a randomly selected neighbour. Assuming *W* is the adjacency matrix of the PPI network and *p*^*t*^ is a vector whose *i*-th element holds the probability of arriving at node *i* at step *t*. Random walk was computed by

(5) $$p^{t+1}=\left(1-r\right)Wp^{t}+rp^{0}$$

where *p*^*0*^ is the initial probability, which is 1/37 for the 37 genes in the KnownSet and 0 for all others; *r* represents the probability of remaining at the same node at the next step. [30] showed that random walk worked robustly against different *r* values, which was also confirmed by our computation (data not shown). *r* was taken to be 0.7 in the current work. For details of random walk approach, see [13].

### Ranking performance comparison

OMIMSet contained 26 known breast cancer genes, CGCSet contained additional 11. The 26 known breast cancer genes in the OMIMSet were used as the KnownSet. Procedure in Figure 1 was followed and the model built, which was then used to rank the 11 known breast cancer genes in the CGCSet. Ranking values in *italic* meant those genes were ranked in the top 10% of the final list. The row of “top 10% average” represented average rankings of those known breast cancer genes in CGCSet that ranked in top 10%, while “all 11 test genes average” represented the average rankings of all 11 genes in the CGCSet (Table 2). In later computation, OMIMSet and CGCSet were combined into a KnownSet of 37 genes.

### Performance evaluation against realistic data noise

**Figure 9:**
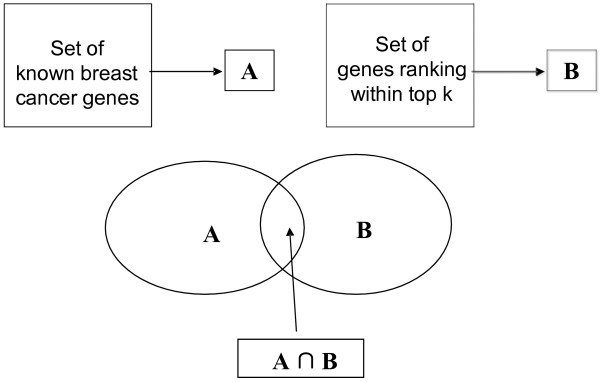
**Computing precision and recall.** A was the set of known breast cancer genes, and B was the set of breast cancer genes which had been predicted as breast cancer genes by the proposed method. Precision represented ability to reject unrelated genes, and recall represented ability to obtain true breast cancer genes.

GOSet was a set of genes enriched with probable breast cancer genes. Its quality was obviously lower than the KnownSet, but higher than a set of random genes nonetheless. To evaluate the proposed method’s robustness with respect to noise in real life data sources, multiples of 37 (the number of known breast cancer genes in the PPI network) genes were sampled from the GOSet (ranging from 0 to 50, with 0 meaning no noise added). Each sample was combined with the KnownSet into an ntegrated source data. Stratified cross-validation was utilized for performance comparison between the proposed approach and the random walk method [13]. The sampled genes were randomly partitioned into five equal subsets, one of the five subsets was retained for testing, and the remaining four subsets were used as training data. The KnownSet was also randomly divided into five equal parts. One of them was combined with one subset of the sampled genes as testing data, the other four with the four remaining subsets of the sample as training data. This procedure was then repeated five times, with each of the five subsets used once as testing data. All results from the five folds were averaged to generate the final result. Performance of the proposed approach and random walk method with respect to data noise were evaluated in terms of the F-score, which was computed from precision and recall (Figure 9). Precision was the fraction of genes ranked within top *k* in the test data that were true known cancer genes; recall was the fraction of known breast cancer genes ranking within top *k*. F-score was then the harmonic mean of precision and recall. *k* was the ranking threshold that was used to decide whether a ranked gene was considered as a predicted positive, that is, genes ranked higher than k were judged as breast cancer genes. The F-score was defined as following,

(6) $$F-\mathrm{score}\left(k\right)=\frac{2\times \mathrm{Precision}\left(k\right)\times \mathrm{Recall}\left(k\right)}{\mathrm{Precision}\left(k\right)+\mathrm{Recall}\left(k\right)}$$

where

(7) $$\mathrm{Precision}\left(k\right)=\frac{\left|A\cap B\right|}{\left|B\right|}$$

(8) $$\mathrm{Recall}\left(k\right)=\frac{\left|A\cap B\right|}{\left|A\right|}$$

where **A** is the number of genes in the KnownSet, **B** is the number of genes ranked within top *k*.

One F-score was computed for each fold, averaging five F-scores (for five-fold cross validation) produced the final F-score.

### Performance evaluation against completely random noise

Random genes were first sampled from the PPI network and added to the KnownSet. Procedure in Figure 1 and random walk computation were then performed. Figure 5 plotted the ratio of random genes to the number of genes in KnownSet (37) and the proportion of added random genes that ranked within top 10%.

## Competing interests

The authors declare that they have no competing interests.

## Authors’ contributions

YN and JY jointly developed the framework. YN collected data. YN and JY performed analyses. YN and JY jointly wrote the manuscript. All authors read and approved the final manuscript.

## Supplementary Material

Additional file 1: Table S1 Supplementary Table 1. Click here for file
